# Correction: The Temporary Incapacity (TI) register as a complementary system to traditional epidemiological surveillance during the COVID-19 pandemic in Spain

**DOI:** 10.1371/journal.pone.0331315

**Published:** 2025-08-29

**Authors:** Dante Culqui Lévano, Sofía Escalona López, Alín Gherasim, Jesús Oliva Domínguez, María Teresa Disdier Rico, Montserrat García Gómez

The affiliation for the first and second authors is not indicated. Dante Culqui Lévano and Sofía Escalona López, are both affiliated with: External Senior Technician (TRAGSATEC). Subdirectorate General for Environmental Health and Occupational Health, Directorate General for Public Health, Ministry of Health, Madrid, Spain.

There are errors in the Author Contributions. The correct contributions are

**Conceptualization:** Dante Culqui Lévano, Montserrat García Gómez

**Data curation:** Dante Culqui Lévano, Jesús Oliva Domínguez, Montserrat García Gómez

**Formal analysis:** Dante Culqui Lévano, Sofía Escalona López, Alín Gherasim, Montserrat García Gómez

**Investigation:** Dante Culqui Lévano, Sofía Escalona López, Alín Gherasim, Montserrat García Gómez

**Methodology:** Dante Culqui Lévano, Sofía Escalona López, Alín Gherasim, Montserrat García Gómez

**Project administration:** Sofía Escalona López, Alín Gherasim, Montserrat García Gómez

**Supervision:** Dante Culqui Lévano, Montserrat García Gómez.

**Validation:** Dante Culqui Lévano, Sofía Escalona López, Jesús Oliva Domínguez, María Teresa Disdier Rico, Montserrat García Gómez.

**Visualization:** Jesús Oliva Domínguez, María Teresa Disdier Rico, Montserrat García Gómez.

**Writing – original draft:** Dante Culqui Lévano

**Writing – review & editing:** Dante Culqui Lévano, Sofía Escalona López, Alín Gherasim, Jesús Oliva Domínguez, María Teresa Disdier Rico, Montserrat García Gómez.

The first author, Dante Culqui Lévano, is incorrectly noted as the corresponding author. The correct corresponding author is Montserrat García Gómez. Montserrat García Gómez’s email address is: mgarciag@sanidad.gob.es.

## References

[1] Lévano DC, López SE, Gherasim A, Domínguez JO, Rico MTD, Gómez MG. The Temporary Incapacity (TI) register as a complementary system to traditional epidemiological surveillance during the COVID-19 pandemic in Spain. PLoS One. 2024;19(5):e0301344. doi: 10.1371/journal.pone.0301344 38768237 PMC11104667

