# Supplementary material for: The Temporary Incapacity (TI) register as a complementary system to traditional epidemiological surveillance during the COVID-19 pandemic in Spain
Source: PLoS One. 2024 May 20;19(5):e0301344. doi: 10.1371/journal.pone.0301344 (PMC11104667; doi:10.1371/journal.pone.0301344)
Supplement: S1 File — (DOCX) [file pone.0301344.s001.docx]

**Response to Editor**

Dear Ms/Mr Editor,

As mentioned above, the Spanish Ministry of Health does not own the Social Security data, so we cannot share any of the data we have used for this work because it is confidential information. These data could be made available upon request if the social security authorises it, after making a request for information on the social security website: <https://sede.seg-social.gob.es/wps/portal/sede/sede/Ciudadanos/2022estadisticas/090316_c_ai?changeLanguage=en>

We are looking forward for your response in case a new Data Availability statement is needed.

Best regards.

Dante Culqui
