# Supplementary material for: The Temporary Incapacity (TI) register as a complementary system to traditional epidemiological surveillance during the COVID-19 pandemic in Spain
Source: PLoS One. 2024 May 20;19(5):e0301344. doi: 10.1371/journal.pone.0301344 (PMC11104667; doi:10.1371/journal.pone.0301344)
Supplement: Attachment — Submitted filename: Response to Reviewers.docx [file pone.0301344.s003.docx]

**Response to Reviewers**

Dear Ms/Mr Editor,

We would like to take this opportunity to thank you for the time dedicated to our article.

We addressed all the comments and suggestions send by the reviewers, and we modified the article accordingly. Below, we are submitting the reviewer´s comments/suggestions and the performed changes:

**Review comments to the Author**

*Please use the space provided to explain your answers to the questions above. You may also include additional comments for the author, including concerns about dual publication, research ethics, or publication ethics. (Please upload your review as an attachment if it exceeds 20,000 characters)*

*Reviewer #1: The paragraph that explains Figure 1, in Results section, remains with incorrect dates in this new version. Please review.*

*Reviewer #2: (No Response)*

**Reply:** We have reviewed all the information in the paragraph with incorrect dates and have included the correct information in the paper.
